# Marine biodegradation of poly[(*R*)-3-hydroxybutyrate-*co*-4-hydroxybutyrate] elastic fibers in seawater: dependence of decomposition rate on highly ordered structure

**DOI:** 10.3389/fbioe.2023.1303830

**Published:** 2023-12-22

**Authors:** Taku Omura, Sakura Tsujimoto, Satoshi Kimura, Akira Maehara, Taizo Kabe, Tadahisa Iwata

**Affiliations:** ^1^ Graduate School of Agricultural and Life Sciences, The University of Tokyo, Bunkyo, Tokyo, Japan; ^2^ Graduate School of Industrial Technology, Nihon University, Narashino, Japan; ^3^ Niigata Research Laboratory, Mitsubishi Gas Chemical Co., Inc., Niigata, Japan

**Keywords:** marine biodegradation, polyhydroxyalkanoate, Poly(3-hydroxybutyrate-*co*-4-hydroxybutyrate), elastic fiber, highly ordered structure, molecular orientation, porosity, biofilms

## Abstract

Here, we report the marine degradability of polymers with highly ordered structures in natural environmental water using microbial degradation and biochemical oxygen demand (BOD) tests. Three types of elastic fibers (non-porous as-spun, non-porous drawn, and porous drawn) with different highly ordered structures were prepared using poly[(*R*)-3-hydroxybutyrate-*co*-16 mol%-4-hydroxybutyrate] [P(3HB-*co*-16 mol%-4HB)], a well-known polyhydroxyalkanoate. Scanning electron microscopy (SEM) images indicated that microorganisms attached to the fiber surface within several days of testing and degraded the fiber without causing physical disintegration. The results of BOD tests revealed that more than 80% of P(3HB-*co*-16 mol%-4HB) was degraded by microorganisms in the ocean. The plastisphere was composed of a wide variety of microorganisms, and the microorganisms accumulated on the fiber surfaces differed from those in the biofilms. The microbial degradation rate increased as the degree of molecular orientation and porosity of the fiber increased: as-spun fiber < non-porous drawn fiber < porous drawn fiber. The drawing process induced significant changes in the highly ordered structure of the fiber, such as molecular orientation and porosity, without affecting the crystallinity. The results of SEM observations and X-ray measurements indicated that drawing the fibers oriented the amorphous chains, which promoted enzymatic degradation by microorganisms.

## 1 Introduction

Currently, 400 million tons of plastics, which are considered essential to our daily lives, are produced annually worldwide, representing a historic growth in production (Plastics Europe, 2016). At least 8 to 11 million tons of this plastic is released into the ocean each year, and if no action is taken, this amount is estimated to increase four-fold by 2050 (World Economic Forum, 2017; The Pew Charitable Trusts and SYSTEMIQ, 2020). In recent years, the negative effects of marine pollution, especially non-biodegradable plastics leaked into the ocean, on marine life have become a global problem. Depending on the location, plastic debris from fishing gear accounts for 10%–90% of marine debris (Lebreton et al., 2018). For example, around Korea, fishing gear generates approximately 75% of marine debris annually (Jang et al., 2014), while in the open ocean, fishing gear accounts for 50%–90% of marine debris (Hammer et al., 2012), most of which is fiber. Fishing nets, fishing lines, ropes, and other fibers used in fishing gear break during use and are lost or abandoned. Subsequently, they unintentionally become entangled with marine creatures and continue to function as fishing gear, known as the ghost fishing cycle (ghost gear). Therefore, to address this problem, there has been active development of plastic fibers that can be completely degraded by microorganisms in the environment into water and carbon dioxide, known as marine biodegradation.

To date, the biodegradability of plastics has been investigated using compost, soil, and river water under aerobic and anaerobic conditions in accordance with methods developed by standardization organizations, such as the International Organization for Standardization (ISO) and the American Society for Testing and Materials International (ASTM International). Compared with these media, the marine environment has fewer microorganisms, resulting in slower or no decomposition (Wang et al., 2021). Therefore, it is impossible to simply extrapolate the results of standard testing to evaluate biodegradability in the marine environment. The marine biodegradation of plastics has been evaluated using seawater and biochemical oxygen demand (BOD)-based tests (Kasuya et al., 1998; Nakayama et al., 2019) as well as field tests at shore (Briassoulis et al., 2019; Lott et al., 2020). These tests also investigate the effects of specific particle size, surface area, and shape on biodegradation (César et al., 2009; Chinaglia et al., 2018; Hino et al., 2023). In addition, polymer crystals (Nishida and Tokiwa, 1992; Nishida and Tokiwa, 1993), lamellar thickness (Koyama and Doi, 1997; Abe et al., 1998), and molecular orientation (Cai et al., 1996; Yoo and Im, 1999; Cho et al., 2003; Fischer et al., 2004; Komiyama et al., 2021) are some parameters that affect the degradation rate of biodegradable plastics. Therefore, the rate of biodegradation could be controlled by adjusting these parameters. To use biodegradable polymers in fiber applications, the polymers must possess enough strength which in turn can be tuned by changing the drawing ratio and the orientation of the molecular chains. However, multiple reports suggest that highly oriented molecular chains of poly[(*R*)-3-hydroxybutyrate] (PHB), poly(*ε*-caprolactone) (PCL), and poly(L-lactic acid) (PLLA) decreases the hydrolysis rate (Mochizuki et al., 1995; Cai et al., 1996; Yoo and Im, 1999; Cho et al., 2003; Fischer et al., 2004; Tsuji et al., 2007). These papers cannot be directly compared because as the orientation degree of the molecular chains increases, the degree of crystallinity also increases significantly, making it unclear which of these parameters affects the degradation rate. In fact, some studies have shown that the microbial degradation rate of biodegradable plastics decrease if the crystallinity is increased without orienting the molecular chains (Nishida and Tokiwa, 1992; Nishida and Tokiwa, 1993).

Polyhydroxyalkanoates (PHAs) are one of the few biodegradable plastics that can be degraded in the ocean, which is a critical issue today, and is attracting particular attention because of its potential to produce high-strength fibers (Iwata et al., 2004). A PHA copolymer that includes a 4HB unit as the second constituent, poly[(*R*)-3-hydroxybutyrate-*co*-4-hydroxybutyrate] [P(3HB-*co*-4HB)], is a promising alternative to non-biodegradable elastic materials owing to its ability to produce fibers with both strength and elasticity, excellent environmental degradability, and biocompatibility/absorbability (Omura et al., 2021; Murayama et al., 2023). The crystals of P(3HB-*co*-4HB) are representative of the Flory exclusion model because the 4HB units are excluded from the crystals formed from 3HB units, and thus the crystallinity saturates at a certain value (Kunioka et al., 1989; Doi et al., 1995). Therefore, even when uniaxial stretching is performed, there is no significant change in crystallinity, indicating that the degree of orientation can be strictly controlled.

PHA crystals have two crystalline phases: an α-form and a β-form (Yokouchi et al., 1973; Orts et al., 1990). In general, when PHA crystallizes, it forms lamellae crystals of the α-form, which is a thermodynamically stable crystalline phase. This form is easily obtained by hot-pressing or casting films. In contrast, the β-form is a stress-induced crystalline phase in uniaxially stretched PHA films (Iwata et al., 2004). In previous research on PHA, researchers have controlled not only the highly ordered structure, such as the melt-cooled α-form and the stress-induced β-form, but also the degree of crystallinity and porosity of the material (Iwata et al., 2003; Tanaka et al., 2007; Kabe et al., 2015; Phongtamrug and Tashiro, 2019; Komiyama et al., 2022). Moreover, studies on enzymatic and environmental degradation (Kumagai et al., 1992; Iwata et al., 1997; Kasuya et al., 1998) have been conducted. However, research on the correlation between these highly ordered structures and environmental degradability remains insufficient. To investigate this relationship, it is necessary to investigate the biodegradation of fibers with controlled highly ordered structures in terms of not only degradation rate but also BOD-curves changes.

The purpose of this study was to investigate the individual effect of uniaxial orientation and porous structure on the microbial degradation rate and BOD-based biodegradation of P(3HB-*co*-16 mol%-4HB), which has a highly ordered structure and is expected to be used in many environmental applications owing to its elasticity. Three types of P(3HB-*co*-16 mol%-4HB) fibers, non-porous as-spun, non-porous drawn, and porous drawn, with different highly ordered structures were prepared by melt spinning, and microbial degradation was performed using seawater from Tokyo Bay. The degradation of each fiber was evaluated using weight measurements, scanning electron microscopy (SEM), wide-angle X-ray diffraction (WAXD), small-angle X-ray scattering (SAXS), gel permeation chromatography (GPC), and BOD testing.

## 2 Experimental study

### 2.1 Materials

P(3HB-*co*-16 mol%-4HB) with a weight-average molecular weight (*M*_w_) of 6.6 × 10^5^ g/mol and polydispersity index (PDI) of 2.9 was provided by Mitsubishi Gas Chemical Co., Japan. P(3HB-*co*-16 mol%-4HB) was obtained in the pure form and used without further purification. The powder (5 g) was dissolved in chloroform (500 mL) and cast into a film.

### 2.2 Processing of non-porous P(3HB-*co*-16 mol%-4HB) fiber

P(3HB-*co*-16 mol%-4HB) fibers were fabricated according to the method described previously (Omura et al., 2021). The cast film of P(3HB-*co*-16 mol%-4HB) (1.5 g) was placed in the furnace of a melt-spinner (IMC-19F8; Imoto Manufacturing Co., Japan) set to 150°C (i.e., lower than the melting temperature of the lamellar crystals), melted for 1 min, and then spun with an extruder rod at a rate of 1 mm/s. A nozzle with a length to diameter (L/D) ratio of 3 and diameter of 1 mm was used, and the fibers were taken up at a rate of 1.8 m/min. The fibers were then stretched by hand until they necked and drawn by five-fold at room temperature. The fibers obtained in this experiment were not heat treated.

### 2.3 Processing of porous P(3HB-*co*-16 mol%-4HB) fiber

P(3HB-*co*-16 mol%-4HB) powder was melt-spun using the same procedure as in 2.2. The melt-spun fibers were taken up at a speed of 1.8 m/min in an ice water bath at 4°C. After isothermal crystallization at 4°C for 72 h, porous fibers were obtained by necking and drawing by 12-fold at room temperature using a manual stretching machine. The fibers obtained in this experiment were not heat treated.

### 2.4 Microbial degradation tests

Seawater used as the source of microbial inoculum was collected from Tokyo Bay: Odaiba Seaside Park (35°37′41.1″N 139°46′16.5″E) in Minato-ku, Tokyo, Japan. Table 1 shows the detailed conditions under which the samples were collected.

**TABLE 1:** Details of the conditions and seawater sampled.

Type	Place	Date	Time	Weather	Temp/°C	Water temp/°C	pH	Viable microorganism counts×10^5^/CFU mL^-1^
Seawater	Tokyo-bay	Mar 2nd	14:00	Cloudy/Rainy	18	15	7.6	2.1

Sediment (soil and sand) was also collected at the same location the natural environmental water (seawater) was collected. Sediment (2 kg) was added to 10 L of the natural water and stirred well. An ethanol-sterilized polyethylene mesh with a 122 µm gap was used to remove impurities, such as leaves, stones, and wood, with stirring. The resulting water was used as the test water.

Degradation tests were performed by adding approximately 20 mg of three P(3HB-*co*-16 mol%-4HB) fibers with different highly ordered structures and 100 mL of test water to each open container. The test was conducted at 25°C with agitation at a rate of 30 rpm for a total of 28 days. Samples were collected every 7 days, ultrasonically cleaned to remove the biofilm from the fiber surface, vacuum dried overnight at room temperature, and weighed for weight loss measurements. For each fiber, three different samples (*n* = 3) were collected every 7 days for weight loss measurements, and the average and deviation were calculated.

### 2.5 Media and culture conditions of bacteria

The total number of viable bacteria in the environmental water was determined using the plate count method (Nishida et al., 1998). Standard agar medium Daigo (23.5 g) and sodium chloride (29.2 g) were added to 1 L of distilled water, and the pH of the solution was adjusted to 7.0 using NaOH (1 mol/L) or HCl (1 mol/L) solution. The solution was autoclaved at 121°C for 15 min and poured into sterile polystyrene petri dishes to prepare the Luria–Bertani (LB) medium. A 10-fold diluted sample solution was prepared by adding 100 μL of collected seawater to 900 μL of sterile artificial seawater (NaCl: 29.2 g/L). This was repeated to prepare 100-, 1,000-, and 10,000-fold diluted sample solutions. Each sample solution (50 µL) was plated on the standard agar medium and incubated at 25°C for 24 h. Colonies formed on the plate were counted, and the number was expressed as colony forming units per mL (CFU/mL).

### 2.6 BOD-based test (BOD-biodegradability)

Biodegradation in the environmental water was evaluated using a BOD measuring device (OxiTop IDS, WTW, and Germany). The BOD test was performed by modifying the method of Suzuki et al. (Suzuki et al., 2017) In a cultivation bottle (internal volume 250 mL), 100 mL of seawater was mixed with 100 µL of buffer solution (Na_2_HPO_4_・2H_2_O 33.3 g/L, K_2_HPO_4_ 21.8 g/L, KH_2_PO_4_ 8.5 g/L, NH_4_Cl 1.7 g/L), 0.5 g/L NH_4_Cl, 0.1 g/L Na_2_HPO_4_, and 5 mg/L allylthiourea. The sample weight was 6-7 mg. BOD tests were conducted in an incubator (25°C) for approximately 1 month, and BOD data were acquired daily. BOD-biodegradability was calculated using Eqs 1, 2:

$$\text{BOD biodegradability }\left(\%\right)=\frac{\text{BO}D_{s}-\text{BO}D_{b}}{\text{ThOD}}\times 100\;$$ (1)

where BOD_s_ (mg) is the BOD value measured when the sample was added, BOD_b_ (mg) is the BOD value measured in blank tests, and ThOD is the theoretical oxygen demand (see Eq. 2).

$$\text{ThOD }\left(\text{mg}\right)=\frac{w\;\left(\text{mg}\right)}{M\;\left(g/\text{mol}\right)}\times \frac{4x+y-2z}{4}\times 32\;\left(g/\text{mol}\right)$$ (2)

where *w* is the initial sample weight (mg), and *M* is the molecular weight of the monomer unit (C_*x*_H_*y*_O_*z*_) (g/mol).

The weight loss was calculated using Eq. 3:

$$\text{Weight loss }\left(\%\right)=\frac{W_{i}-W_{f}}{W_{i}}\times 100$$ (3)

where *W*_*i*_ is the initial sample weight (mg) and *W*_*f*_ is the sample weight after microbial degradation (mg).

### 2.7 Characterization

#### 2.7.1 Scanning electron microscopy

The surface morphology of the fibers was observed using a scanning electron microscope (JCM-7000, JEOL, and Japan) operated at an accelerating voltage of 5 kV. Two different methods were used to treat the fibers after microbial degradation to obtain fibers with and without adhered bacteria. First, the collected fibers were soaked overnight in a 3% sodium chloride formaldehyde solution, and samples with microorganisms attached to the fiber surface were freeze-dried after solvent displacement with ethanol/*t*-butanol and then analyzed with SEM. Second, the collected samples were ultrasonically cleaned to remove the biofilm adhering to the fiber surface and vacuum dried overnight at room temperature. These samples were gold-coated using an ionic stepper (MSP-1S, Vacuum Device Co., Japan) before observation.

#### 2.7.2 Gel permeation chromatography

The molecular weights (*M*_w_ and number-average, *M*_n_) and PDI of the samples were measured using gel permeation chromatography (GPC; RID-20A differential refractive index detector, Shimadzu). Samples in chloroform were passed through columns (K-806M, K-802) with a flow rate of 0.8 mL/min at 40°C. A calibration curve was prepared using polystyrene (PS) standards (Shodex).

#### 2.7.3 X-ray diffraction

Two-dimensional (2D) WAXD measurements were performed using a Micromax-007HF system (Rigaku, Japan) equipped with a CuKα irradiation source (λ = 0.15418 nm, operated at 40 kV and 30 mA), imaging plate reader (RAXIA-Di, Rigaku, Japan), and imaging plates (BAS-SR 127, 2,540 × 2,540 pixels, 50 × 50 μm2/pixel, Fujifilm Corporation, Japan). The distance between the sample and camera was 83 mm, and the sample and detector were placed in a vacuum chamber at room temperature. Single fibers were measured with an irradiation time of 5 min. The obtained 2D-WAXD diffractograms were converted to 1D-WAXD patterns using the 2DP software (Rigaku) to determine the crystallinity *X*_c_ and crystal orientation *f*_(020)_.

2D SAXS measurements were performed on the BL03XU beamline at the SPring-8 synchrotron radiation facility (Harima, Japan) using an X-ray wavelength of 0.1 nm, and 2D diffractograms were recorded on an array detector (PILATUS3 S 1M, Rigaku). The distance between the sample and camera was 2,278 mm, and silver behenate (BeAg) was used as the calibration sample. The sample and detector were placed at room temperature and atmospheric pressure. Single fiber measurements were performed with an irradiation time of 1 s. The obtained 2D-SAXS images were analyzed using the MDIP software. The long period, *L*_p_ was obtained by analyzing the meridional region of the SAXS pattern.

## 3 Results and discussions

### 3.1 Details of P(3HB-co-16 mol%-4HB) fibers

Details of the three types of P(3HB-co-16 mol%-4HB) fibers prepared are shown in Table 2. The weight of the fibers used in the degradation tests with seawater was approximately 20 mg, and the corresponding fiber length and diameter are shown in Table 2. As-spun and 5-fold drawn fibers were densely packed with polymer chains inside the fiber. In contrast, fibers drawn by 12-fold after 72 h of isothermal crystallization at 4°C had numerous discontinuous pores of approximately 5 μm inside the fiber, which was equivalent to a porosity of 45%. The crystals of P(3HB-*co*-4HB) are representative of the Flory exclusion model, in which 4HB units are excluded from crystals formed from 3HB units, and thus the crystallinity saturates at a certain value (Kunioka et al., 1989; Doi et al., 1995). This suggests that crystallization of 3HB unit is inhibited by the 4HB unit randomly incorporated during copolymerization, and thus crystallization can be controlled by drawing and heat treatment. Moreover, it is possible to control the degree of orientation without affecting the degree of crystallinity by drawing and heat treatment, thus obtaining samples with different degrees of orientation only. This enables a comparison of the samples without considering the effect of crystallinity. The 2D-WAXD image (Supplementary Figure S1A) of the as-spun fiber showed a ring pattern, indicating that the crystals were randomly distributed and unoriented, with a crystallinity of 41%. In contrast, the 2D-WAXD images (Supplementary Figures S1F, K) of both drawn fibers (non-porous and porous) indicated that the fibers were oriented in the α-form, with an orientation of 0.9 and crystallinity of 36% and 38%, respectively. In the previously reported uniaxial drawing of PCL, the crystallinity increases from 40% to 64% with drawing, and therefore it was impossible to simply attribute the enzymatic degradation rate to either the molecular orientation or the crystallinity (Mochizuki et al., 1995). However, in this study, the crystallinity of the P(3HB-co-16 mol%-4HB) elastic fibers did not change significantly before and after drawing, and thus the effect of orientation only on the degradation rate could be evaluated. In addition, the effect of the internal pores on the degradation rate could be evaluated.

**TABLE 2:** Detail of various P(3HB-co-16 mol%-4HB) elastic fibers with different higher-ordered structure.

	As spun	Non-porous	Porous
Overall pictures	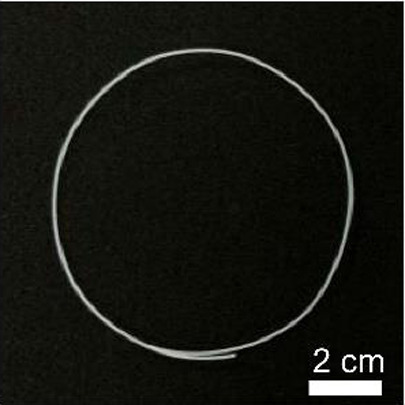	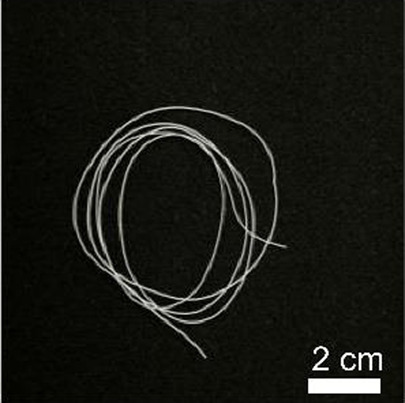	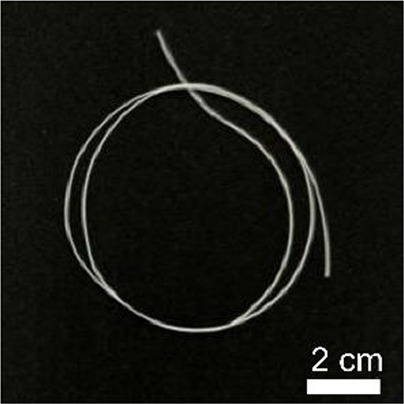
Cross section, SEM Image	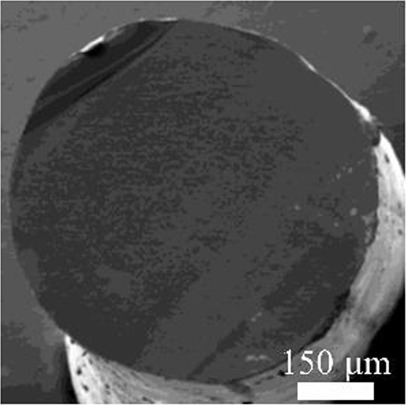	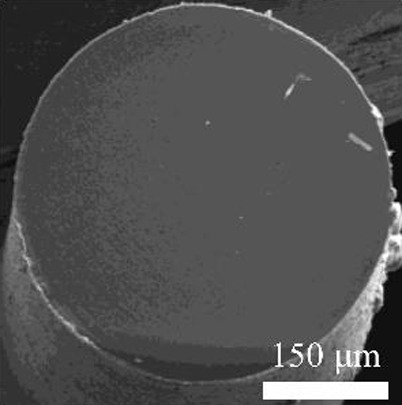	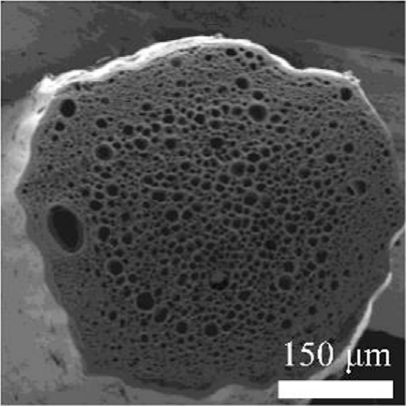
Draw ratio, λ	1	5	12
Crystallinity, X_c_/%	41	36	38
(020) orientation, f	-	0.9	0.9
Weight/mg	15–19	18–20	15–19
Diameter/μm	850	312	364
Length/cm	2.2	14.1	16.1

### 3.2 Effect of highly ordered structure on marine biodegradability of P(3HB-*co*-16 mol%-4HB) fibers

To investigate the effect of morphology on the degradation rate, the weight losses of three types of P(3HB-*co*-16 mol%-4HB) fibers (non-porous as-spun, non-porous drawn, and porous drawn) immersed in seawater from Tokyo Bay were evaluated. After 28 days of microbial degradation in seawater from Tokyo Bay, the weight losses of the two types of drawn fibers (non-porous and porous) were approximately 100%, indicating good marine degradability (Figure 2). In the case of the porous drawn fiber, the internal porous surfaces were exposed as the smooth external surfaces were degraded by the microorganisms, further increasing the exposed surface area, which likely contributed to the fast marine degradation rate (Figure 4, discussed later). In contrast, the as-spun fibers were not completely degraded in 28 days, and the weight loss after 28 days was only 46%. In this study, the weight of the fibers used was standardized to approximately 20 mg in order to make comparisons by weight loss. Fiber length and fiber surface area differ due to the thicker fiber diameter of the as-spun fiber (850 μm) compared to the fiber diameter of the two drawn fibers (about 300 μm). Since microbial degradation generally proceeds from the surface, the rate of biodegradation is strongly influenced by the surface area of the sample. Therefore, microbial degradation of samples with different surface areas must be evaluated by degradation rates considering the surface area. The degradation rate was 3.3 mg/cm^2^/week for the as-spun fiber, 5.1 mg/cm^2^/week for the drawn fiber (non-porous) and 5.5 mg/cm^2^/week for the drawn fiber (porous), indicating that the degradation rate for the drawn fibers (non-porous and porous) was about 1.5 times faster than that for the as-spun fiber.

### 3.3 Effect of P(3HB-*co*-16 mol%-4HB) morphology on BOD biodegradation

**FIGURE 1:**
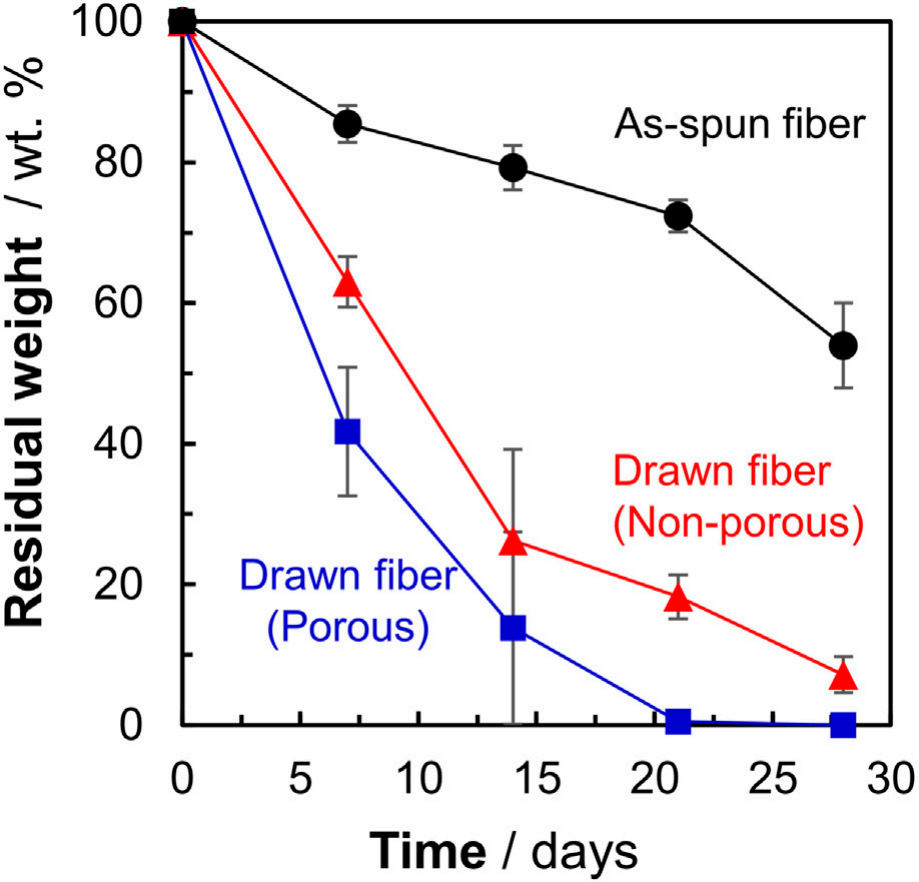
Weight losses of P(3HB-*co*-16 mol%-4HB) fibers with different highly ordered structures. Black: as-spun, red: non-porous drawn, blue: porous drawn. Filled black circles: as-spun, red triangles: non-porous drawn, blue squares: porous drawn.

**FIGURE 2:**
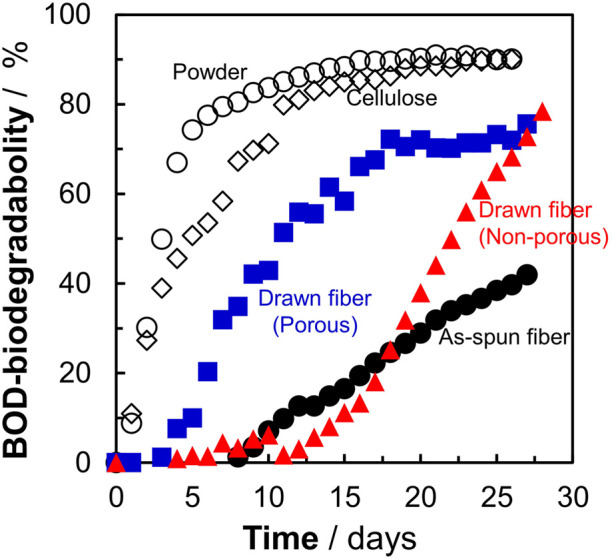
Effect of sample morphology on BOD-biodegradability of P(3HB-co-16 mol%-4HB) fibers in seawater from Tokyo Bay. Open circles : P(3HB-*co*-16 mol%-4HB) powder, open diamonds: reference cellulose, filled black circles: as-spun, red triangles: non-porous drawn, blue squares: porous drawn.

Figure 1 shows that the P(3HB-*co*-16 mol%-4HB) fibers lost weight in seawater. This indicated that the P(3HB-*co*-16 mol%-4HB) fibers were hydrolyzed by microorganism-secreted enzymes into substances with low molecular weights and high water solubility. However, weight loss is not equivalent to complete biodegradation into water and carbon dioxide. Therefore, the BOD test was conducted to confirm that the fibers were broken down into water and carbon dioxide by the microorganisms. The shape of the sample is reported to have a significant effect on the BOD test (Komiyama et al., 2021; Hino et al., 2023). It can be assumed that the faster the rate of enzymatic degradation by microorganisms to low molecular weight compounds (the faster the weight loss), the steeper the BOD-biodegradability curve. Here, in addition to the reference, P(3HB-*co*-16 mol%-4HB) powder, three types of P(3HB-*co*-16 mol%-4HB) fibers (as-spun non-porous fiber, drawn non-porous fiber, and drawn porous fiber), with evaluated microbial degradability by weight loss (Figure 1), were used to investigate the effect of shape on the BOD test (Figure 2). For P(3HB-*co*-16 mol%-4HB) powder and the two types of drawn fibers, which showed 100% weight loss in 28 days, BOD-biodegradability was approximately 70%–90% or more in 28 days. This indicated complete biodegradation because the rest of about 10%–30% is considered to be used for biomass formation in microorganism cells (Ohura et al., 1999). The rise of the BOD curve during the period in which low-molecular-weight compounds are completely degraded by microorganisms into carbon dioxide depends on the shape of the sample. The rise was steepest for the powder and flattened in the order of porous drawn fiber (with the fastest weight loss rate) > non-porous drawn fiber > non-porous as-spun fiber. As shown, the shape and highly ordered structure of the fibers had a remarkable effect on BOD biodegradation. In addition, the BOD curves obtained for the three fiber types did not rise in the early stage of the BOD test (5–10 days) because the surface area of the fiber was smaller than that of the powder, and it took time for the microorganisms on the fiber surface to form a biofilm and oligomerize through enzyme secretion and enzymatic hydrolysis.

### 3.4 Microorganisms on fibers after biodegradation

**FIGURE 3:**
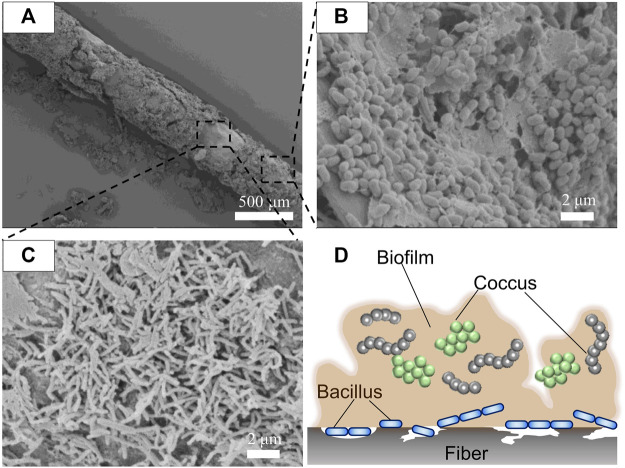
SEM images of **(A)** P(3HB-*co*-16 mol%-4HB) drawn elastic fiber (porous) with the biofilm attached after biodegradation in seawater for 7 days, **(B)** microorganisms present in the biofilm, and **(C)** microorganisms on the fiber surface. **(D)** Schematic model of microorganisms in the plastisphere.

To confirm whether the P(3HB-*co*-16 mol%-4HB) fibers were degraded by microorganisms, the fibers were soaked in seawater and then treated with formaldehyde without washing for SEM observation. After 7 days, the P(3HB-*co*-16 mol%-4HB) fiber surface was covered with a biofilm (Figure 3A). This indicated that the P(3HB-*co*-16 mol%-4HB) fibers were not degraded physically but by microorganisms. Interestingly, different forms of microorganisms were accumulated inside the biofilm (Figure 3B) and on the fiber surface where the biofilm had not formed (Figure 3C). Round-shaped bacteria (cocci) were observed inside the biofilm, while rod-shaped bacteria (elongated bacilli) were found on the fiber surface where no biofilm had formed (Figure 3D). In general, depolymerase-secreting microorganisms that enzymatically degrade polymers may not always metabolize water-soluble low-molecular-weight substances. In contrast, microorganisms that do not participate in enzymatic degradation may metabolize low-molecular-weight compounds. The diverse microorganisms observed in the fibers after degradation suggested that the P(3HB-*co*-16 mol%-4HB) plastisphere was composed of diverse polyester degraders and microorganisms that metabolized the byproducts of enzymatic degradation, stabilized the biofilm, and preyed on other microorganisms, meaning that they coexisted.

### 3.5 Morphological changes of P(3HB-*co*-16 mol%-4HB) fibers during marine biodegradation

**FIGURE 4:**
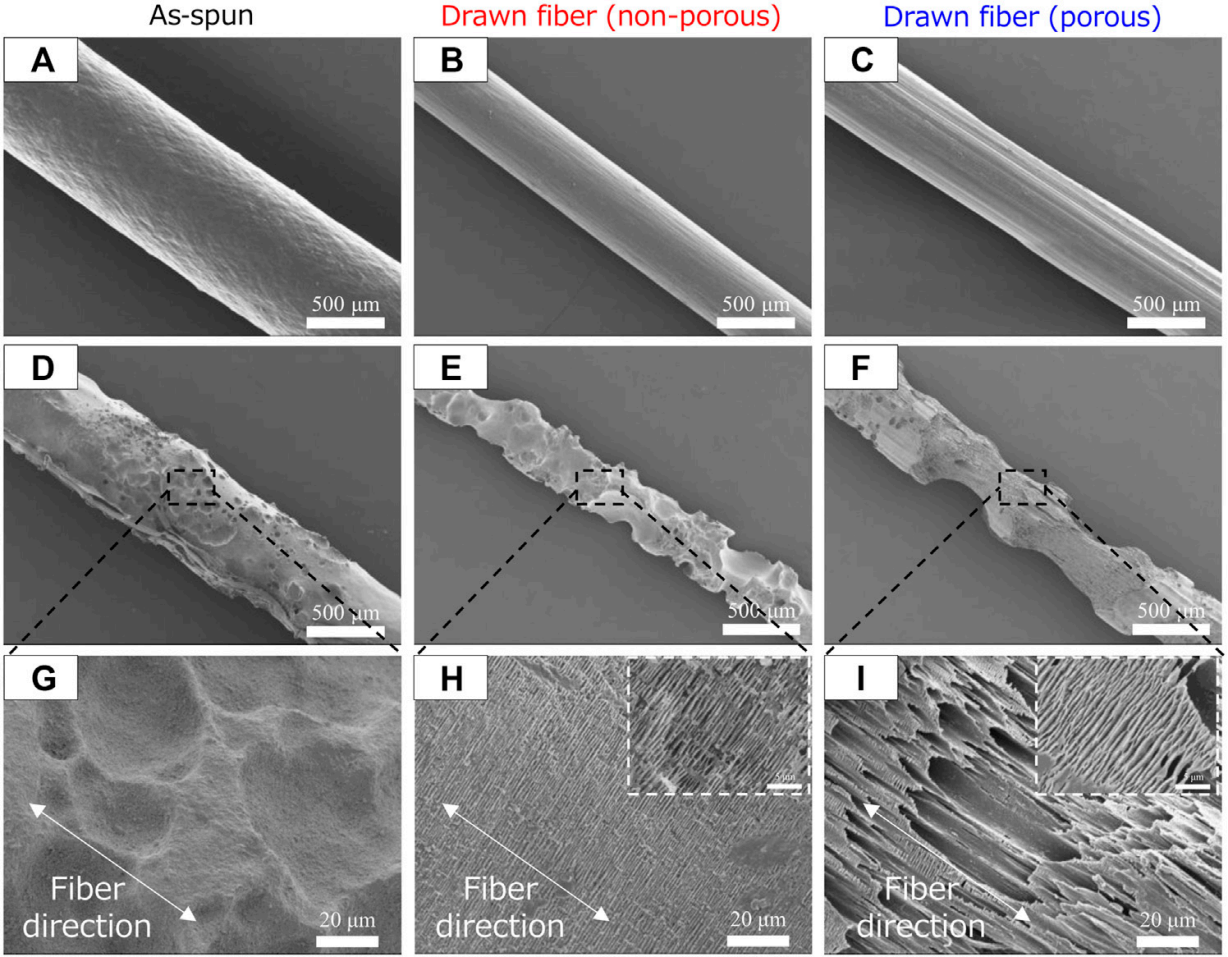
SEM images of P(3HB-*co*-16 mol%-4HB) fibers before **(A–C)** biodegradation and **(D–I)** after 1 week in seawater from Tokyo Bay: **(A, D, G)** asspun, **(B, E, H)** non-porous drawn, and **(C, F, I)** porous drawn.

**FIGURE 5:**
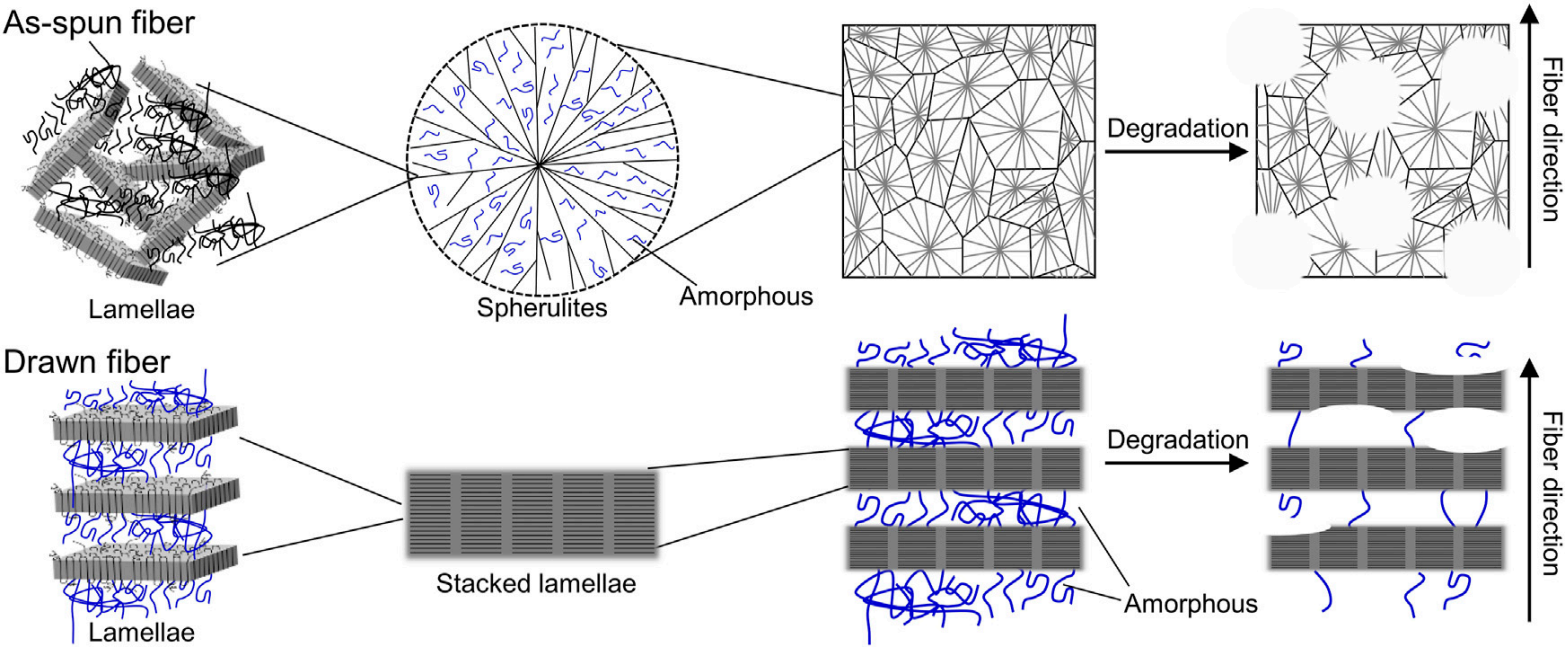
Illustration of assumed biodegradation patterns of fibers with different higher-order structures.

SEM images of the fiber surfaces showed that all ultrasonically washed fibers were degraded by microorganisms (Figures 4A–F). After microbial degradation, the surface of the as-spun fiber was rough and irregular, indicating that microbial biodegradation proceeded randomly (Figure 4G). In contrast, the two types of drawn fibers had a stacked lamellar structure perpendicular to the stretch direction (Figures 4H, I). Illustration of assumed biodegradation patterns of fibers with different higher-order structure are shown in Figure 5. This suggested that the lamellar crystals were oriented by drawing and biodegradation proceeded from the amorphous region of the fiber (Omura et al., 2021). This is because the amorphous region of polymers is less dense and thus more susceptible to attack than the crystalline region. As Abe et al. has reported that the erosion rate of amorphous phase is much larger than that of crystalline phase (Abe et al., 1998). By drawing and orienting the fibers, the amorphous chains are also oriented, and enzymatic degradation by microorganisms can easily proceed from the amorphous chains. Therefore, the biodegradation rate of the drawn fibers may be faster than that of the as-spun fiber. Furthermore, in the porous drawn fiber, the numerous pores inside the fiber are exposed, which increases the surface area. For these reasons, among the fibers investigated, the porous drawn fiber exhibited the fastest degradation rate (Figure 1). The results indicated that it was possible to increase the rate of marine degradation by changing the highly ordered structure of the fibers, such as fiber orientation and porous structure.

### 3.6 Changes in crystallinity with biodegradation

**FIGURE 6:**
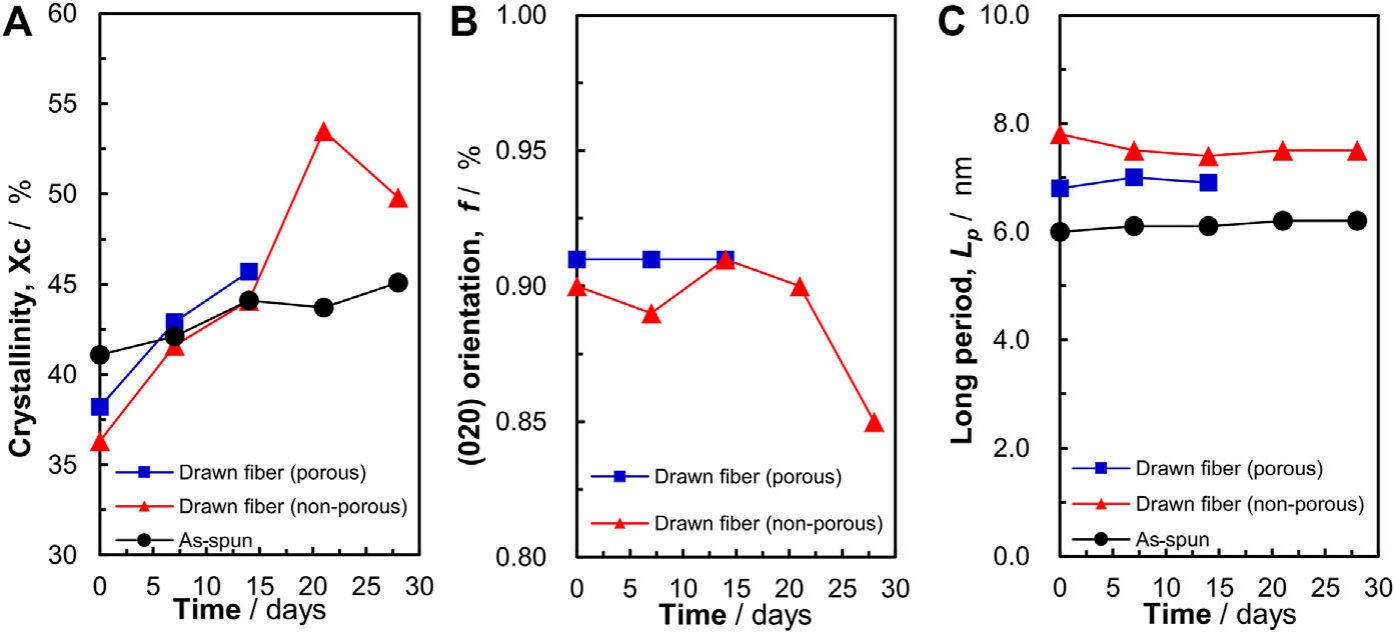
Changes in the **(A)** crystallinity, **(B)** (020) orientation, and **(C)** long period of P(3HB-*co*-16 mol%-4HB) fibers resulting from biodegradation. Black: as-spun, red: non-porous drawn, blue: porous drawn.

As shown in Figure 4, P(3HB-*co*-16 mol%-4HB) fibers were decomposed from the amorphous part. Therefore, we evaluated the P(3HB-*co*-16 mol%-4HB) fibers after degradation using WAXD and SAXS measurements (Supplementary Figures S1, S2). In the 2D-WAXD image of the as-spun fiber before biodegradation, ring diffraction of randomly distributed crystals was observed (Supplementary Figure S1A). In contrast, in the images of the two types of drawn fibers (non-porous and porous fibers) before decomposition, diffraction of lamellar crystals derived from the α-form oriented with the fiber axis was observed (Supplementary Figures S1F, K). Notably, for all fibers, only diffraction of lamellar crystals composed of the α-form was observed, and diffraction of the β-form in a planar zigzag structure was not observed. Iwata et al. reported that for P(3HB) fibers with two types of molecular chains, α-form and β-form, which are controlled and oriented, enzymatic degradation of the β-form occurs at a faster rate than that of the α-form (Iwata et al., 2006). However, because the β-form was not observed in the fibers used for the biodegradation tests in this study, we discuss the degradation of the crystalline (α-form) and amorphous region. For all fibers, the crystallinity tended to increase with degradation time (Figure 6A). This indicated that the relative degree of crystallinity increased because biodegradation proceeded from the amorphous region, and thus the crystalline region remained intact. After biodegradation, the crystallinity of the as-spun fiber did not change as much as that of the two types of drawn fibers (non-porous and porous). This is because the amorphous and crystalline regions of the as-spun fiber are randomly decomposed, whereas the amorphous regions of the drawn fibers are preferentially decomposed, as shown in Figures 4H, I. After 20 days of biodegradation, the crystallinity of the non-porous drawn fiber decreased, which was attributed to the decomposition of the lamellar crystals as well as the progressive decomposition of the amorphous region. The crystal orientation remained almost constant during decomposition, and the orientation decreased just before the end of decomposition (Figure 6B). This suggested that the amorphous molecules that held the lamellae or stacked lamellae in place were decomposed, which destabilized and rotated the lamellae or stacked lamellae. Similarly, the long period remained constant as decomposition proceeded (Figure 6C). Yoo et al. reported a similar trend for the hydrolysis of PCL (Yoo and Im, 1999), suggesting that the crystals remain oriented to some extent, irrespective of microbial degradation.

### 3.7 Changes in molecular weight with biodegradation

**FIGURE 7:**
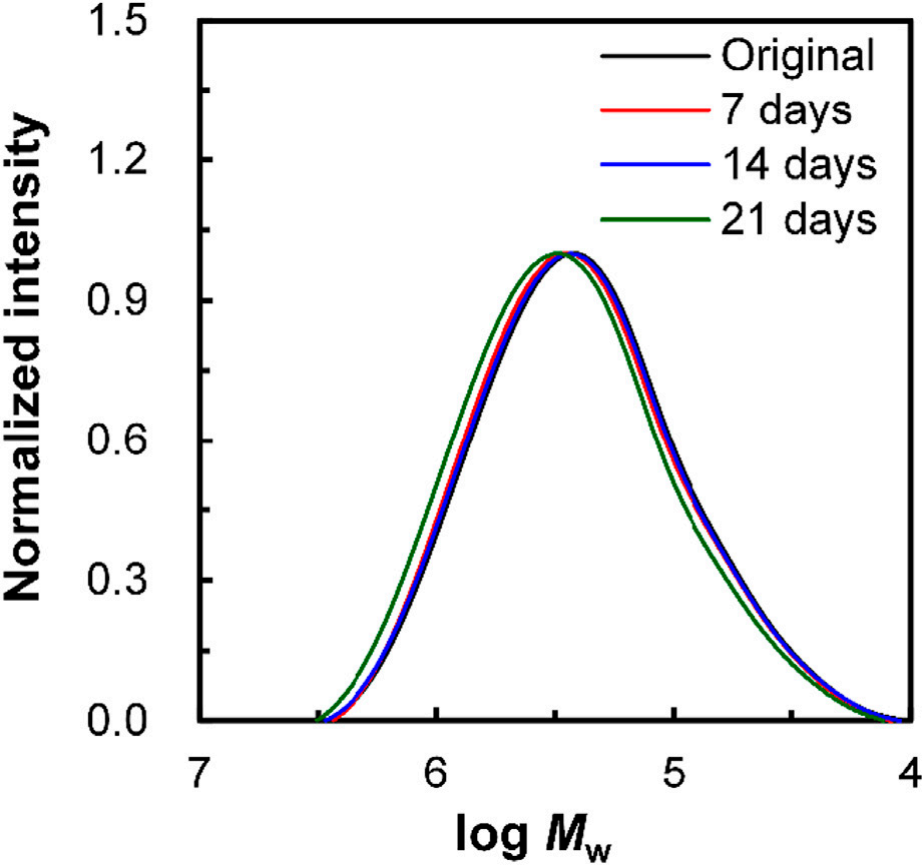
Change in the molecular weight, *M*_w_, of non-porous drawn P(3HB-*co*-16 mol%-4HB) fiber resulting from biodegradation. Black: original fiber, red: 7 days, blue: 14 days, and green: 21 days.

To confirm whether enzymatic hydrolysis proceeded via surface or bulk degradation, the molecular weight of porous drawn P(3HB-*co*-16 mol%-4HB) fibers was determined (Figure 7). The molecular weight did not change before and after the degradation test, suggesting that microbial degradation progressed from the surface. Surface decomposition proceeding intensively on the surface of the material produces a continuous succession of water-soluble low-molecular-weight compounds. In addition, degradation by hydrolysis requires enzymes secreted by microorganisms, which cannot reach the inside of the material owing to their size, and thus degradation proceeds only on the surface of the material (Tsuji and Ishida, 2002). Von Burkersroda et al. reported that the degradation mechanism of biodegradable plastics changes from bulk degradation to surface degradation when the thickness exceeds a critical value (*L*_critical_) (Von Burkersroda et al., 2002). Considering that the fibers used in this study exceeded the critical value (fiber diameter >100 μm), the enzymatic degradation of P(3HB-*co*-16 mol%-4HB) fibers in seawater from Tokyo Bay likely proceeded by surface degradation. In addition, we found that the highly ordered structure of the fiber surface had a significant effect on the degradation rate.

## 4 Implication

These results provide information not only for controlling the degradation rate of biodegradable plastics, but also for future internationally standardized biodegradability tests. The P(3HB-*co*-16 mol%-4HB) elastic fiber prepared in this study shows not only excellent marine biodegradability but also bio-absorbability, and is expected to be applied to surgical sutures. Even in the case of use as surgical sutures, which are temporary fixative materials, the results obtained in this study may be used to fabricate various fibers with controlled duration (rate) of decomposition *in vivo*.

## 5 Conclusion

In this study, the effect of different highly ordered structures on the marine degradability of P(3HB-*co*-16 mol%-4HB) elastic fibers was investigated. The environmental degradation of P(3HB-*co*-16 mol%-4HB) fibers in seawater from Tokyo Bay was evaluated. The biodegradation rates of the three types of P(3HB-*co*-16 mol%-4HB) fibers (non-porous as-spun, non-porous drawn, and porous drawn) were different and decreased in the order of porous drawn > non-porous drawn > non-porous as-spun. BOD tests revealed that the P(3HB-*co*-16 mol%-4HB) elastic fibers were completely biodegraded by microorganisms in the ocean, which was significantly influenced by the morphology of the fibers. Microorganisms adhered to the fiber surface during degradation, suggesting that microorganisms coexisted and decomposed the fiber, indicative of microbial degradation. The SEM images showed that fibers oriented by drawing had a stacked lamellar structure perpendicular to the direction of drawing after the degradation test. In addition, X-ray analysis revealed that the crystallinity of the fibers increased after degradation, suggesting that enzymatic degradation of biodegradable plastics proceeded from the amorphous region, which was easily promoted by increasing the orientation of the molecular chains.

## Data Availability

The original contributions presented in the study are included in the article/Supplementary Material, further inquiries can be directed to the corresponding author.
