# Supplementary material for: Marine biodegradation of poly[(R)-3-hydroxybutyrate-co-4-hydroxybutyrate] elastic fibers in seawater: dependence of decomposition rate on highly ordered structure
Source: Front Bioeng Biotechnol. 2023 Dec 22;11:1303830. doi: 10.3389/fbioe.2023.1303830 (PMC10766686; doi:10.3389/fbioe.2023.1303830)
Supplement: Supplementary file 1 [file DataSheet1.PDF]

Supplementary Material

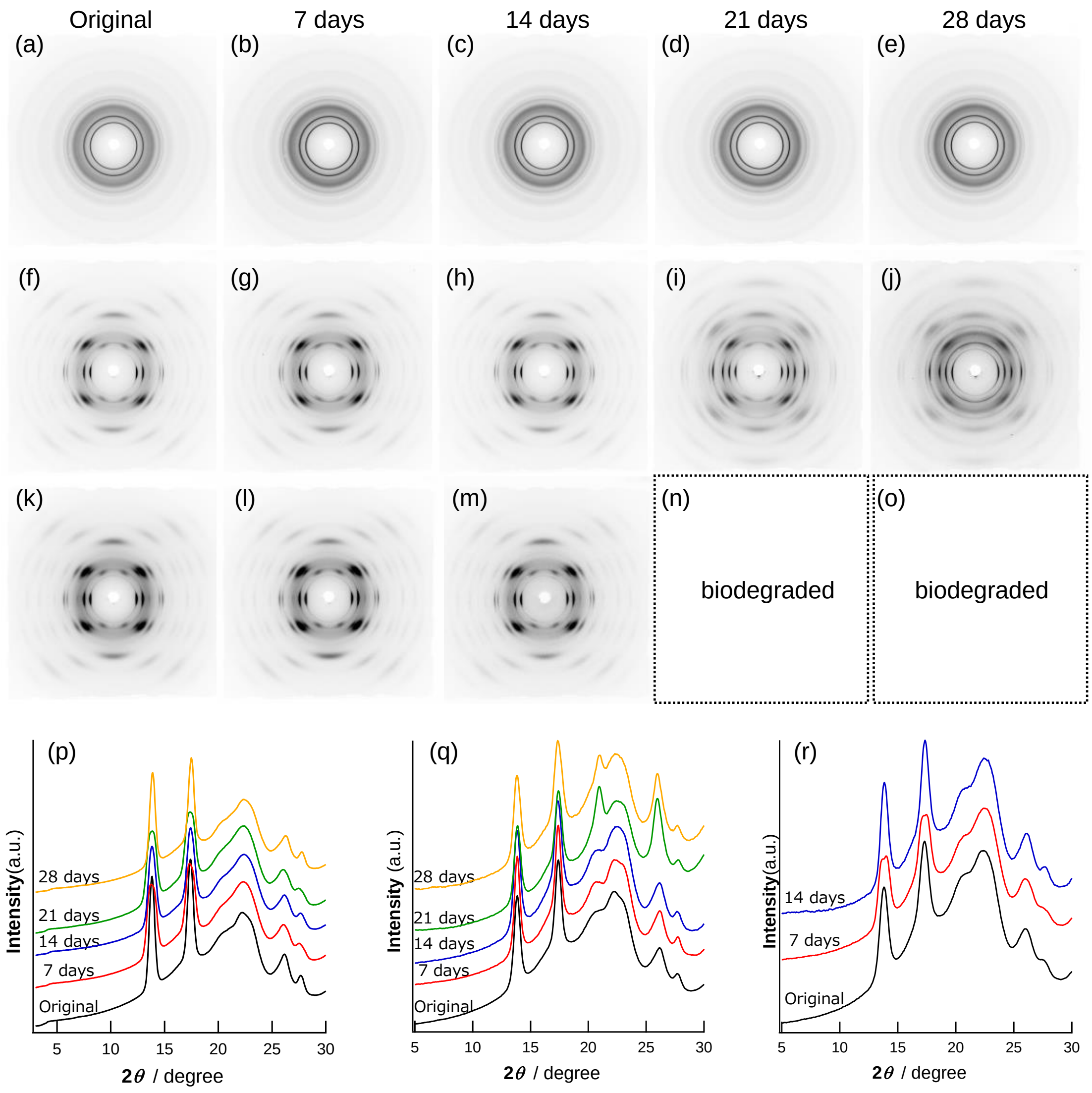

Fig. S1. 2D-WAXD images of P(3HB-*co*-16 mol%-4HB) fiber (a~e) as spun, (f~j) non-porous (λ=5) and (k~o) porous (λ=12) and its 1 D-WAXD profiles of (p) as spun, (q) non-porous (λ=5) and (r) porous (λ=12) after biodegradation with seawater from Tokyo-bay.

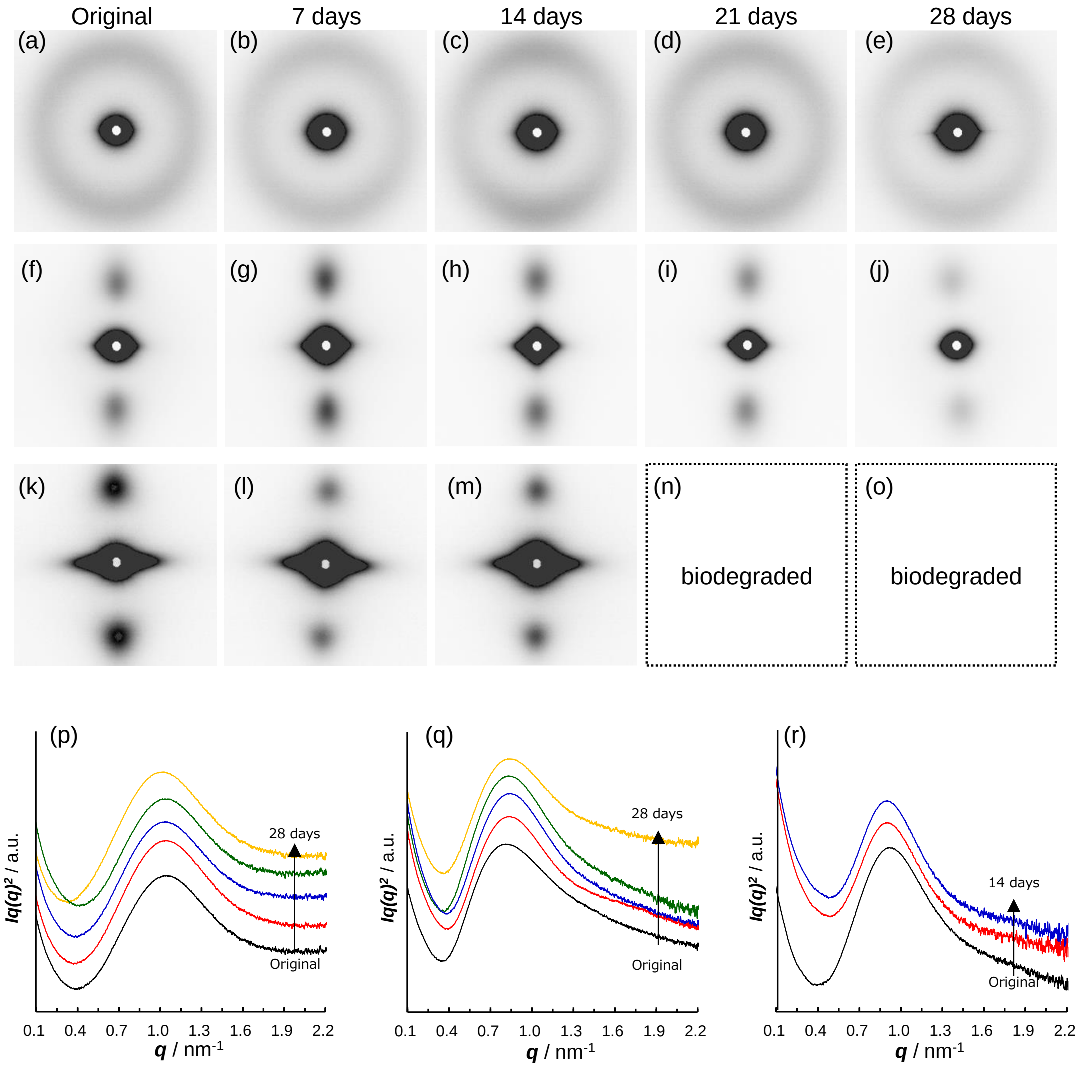

Fig. S2. 2D-SAXS images of P(3HB-*co*-16 mol%-4HB) fiber (a~e) as spun, (f~j) non-porous (λ=5) and (k~o) porous (λ=12) and its 1 D-SAXS profiles of (p) as spun, (q) non-porous (λ=5) and (r) porous (λ=12) after biodegradation with seawater from Tokyo-bay.
